# Resource in Emergency

**Published:** 1901-01-19

**Authors:** 


### RESOURCE IN EMERGENCY.

Few things are more shocking than sudden death during the adminstration of an anæsthetic, and seldom is the nerve of a medical man more severely tried than when, hurried and flurried as he may be by rushing to a case, he has to decide on the spur of the moment what to do, and has to act upon his decision without the loss of a moment's time. A case has recently been reported which well illustrates the extent to which the life of a patient might hang upon the immediate action of the doctor, and how the slightest momentary lack of initiative and resource might turn the scale in the wrong direction. An apparently healthy miner went to a dentist to have a number of teeth extracted. The dentist administered nitrous oxide gas. The removal of the teeth was successfully accomplished, and sponges, without attachment outside the mouth, were used to press on the gums to check the hæmorrhage. The deceased sat listlessly in the chair for two or three minutes after the completion of the operation, when, in the words of the account given in the *Lancet*, he " sprang to his feet, drew in a deep breath, and gasped for air." The dentist at once felt for any obstruction in the air passages, and, finding none, he commenced artificial respiration, and a medical man was sent for. Now here is the lesson. The doctor quickly arrived and " found the patient deeply cyanosed, lying upon the floor, and the dentist performing artificial respiration. The pupils were widely dilated, and life appeared to be extinct." Probably it was extinct. Probably there was nothing to be done, and possibly all that the doctor did—pulling forward the tongue, again employing artificial respiration, and examining for any foreign body which might be lodged in the air passages—was done on a dead man. Possibly, however, it was not; and if not, what a chance of distinguishing himself that medical man lost, and of carrying down to his dying day the sweet memory of having saved a life! For on a *post-mortem* examination a piece of sponge was found lodged below the vocal cords, and if before life finally took wings a scalpel or a pocket-knife could have been plunged into the windpipe, or if the crico-thyroid membrane could have been slit, and if, say by turning a hairpin in the wound, the opening could have been kept free for air to enter, perchance even that man might now have been alive. We do not say that such a result was possible; for to fetch a doctor takes time, and those few minutes may with a struggling man prove to be beyond the utmost limit through which airless life can be maintained. But, for all that, this miserable accident teaches the necessity of ready resource and of rapid action in the presence of emergencies.

